# Developing a European Psychotherapy Consortium (EPoC): Towards Adopting a Single-Item Self-Report Outcome Measure Across European Countries

**DOI:** 10.32872/cpe.13827

**Published:** 2024-09-30

**Authors:** Miguel M. Gonçalves, Wolfgang Lutz, Brian Schwartz, João Tiago Oliveira, Suoma E. Saarni, Orya Tishby, Julian A. Rubel, Jan R. Boehnke, Adrian Montesano, Dario Paiva, Davide Ceridono, Emmanuelle Zech, Jochem Willemsen, Samuli I. Saarni, Katarina Kompan Erzar, Luís Janeiro, Omar C. G. Gelo, Paula Errázuriz, Pawel Holas, Rafał Styła, Tatjana Rožič, Tom Rosenström, Vera Békés, Zsolt Unoka, Michael Barkham

**Affiliations:** 1 CIPsi – Psychology Research Center, School of Psychology, University of Minho, Braga, Portugal; 2 Department of Psychology, University of Trier, Trier, Germany; 3 Helsinki University Hospital, University of Helsinki, Helsinki, Finland; 4 Department of Psychology, Hebrew University, Jerusalem, Israel; 5 Department of Psychology, Osnabrück University, Osnabrück, Germany; 6 School of Health Sciences, University of Dundee, Dundee, United Kingdom; 7 Faculty of Psychology and Educational Sciences, Universitat Oberta de Catalunya, Barcelona, Spain; 8 Institute for Research on Intrapsychic and Relational Processes, IRPIR, Rome, Italy; 9 Université Catholique de Louvain, Ottignies-Louvain-la-Neuve, Belgium; 10 University of Ljubljana, Ljubljana, Slovenia; 11 University of Algarve, Faro, Portugal; 12 University of Salento, Salento, Italy; 13 Pontificia Universidad Católica de Chile / PsiConecta Mental Health NGO, Santiago, Chile; 14 University of Warsaw, Warsaw, Poland; 15 Sigmund Freud University Vienna – Ljubljana branch, Ljubljana, Slovenia; 16 Yeshiva University, New York, USA; 17 Semelweis Unversity, Hungarian Cognitive Behavior Therapy Association, Budapest, Hungary; 18 Clinical and Applied Psychology Unit, School of Psychology, University of Sheffield, Sheffield, United Kingdom; Philipps-University of Marburg, Marburg, Germany

**Keywords:** psychological therapies, European Psychotherapy Consortium, EPoC, practice-based evidence, routine outcome monitoring

## Abstract

**Background:**

Complementing the development of evidence-based psychological therapies, practice-based evidence has developed from patient samples collected in routine care, addressing questions relevant to patients and practitioners, and thereby expanding our knowledge of psychological therapies and their impact. Implementation of assessments in routine care allows for timely clinical decision support and the collection of multiple practice-based data sets by addressing the needs of patients and clinicians (e.g., routine outcome monitoring) and the needs of researchers (e.g., identifying the impact of therapist variables on outcomes).

**Method:**

In this article we describe an initiative developed in Europe, through the European Chapter of the Society for Psychotherapy Research, aimed at creating a consortium that has the potential for collecting data on tens of thousands of patients per year.

**Results:**

A survey identified one of the main problems in the development of a common data set to be the heterogeneity of measures used by members (e.g., 87 different pre-post outcomes). We report on the results of the survey and the initial stage of identifying a single-item – the Emotional and Psychological Outcome (EPO-1) – measure and the process of its translation into multiple European languages.

**Conclusions:**

We conclude this first stage of the overall project by discussing the future potential of the Consortium in relation to the development of procedures that allow crosswalks of outcome measures and the creation of a task force that may be consulted when new data sets are collected, aiming for new common measures to be implemented and shared.

## Highlights

- Many clinics in Europe collect patient data and assess outcomes using different measures.
- Developing common metrics across different countries would facilitate data sharing and analyses.
- An existing single item, measuring patient emotional and psychological outcomes (EPO-1), was adopted.
- The EPO-1 has been translated into 11 languages, to date, and is being used in many countries.

Over the past two decades, a complementary paradigm to evidence-based practice has developed in the form of practice-based evidence as a means of enhancing the overarching evidence-base of psychological therapies (Barkham & Lambert, 2021; Castonguay et al., 2021; Lutz et al., 2021). While the former concentrates on treatments and techniques using the methodologies of randomized controlled trials (RCTs) and meta-analyses, the latter aims to systematically collect patient data in routine clinical settings in connection with specific treatment goals or desired outcomes. However, data administration, management, and processing of outcome measures in routine practice are time consuming and a burden to under-funded services. In response to aspirations to collect data but also reduce administrative demands, there has been a move towards the development and adoption of relatively brief outcome measures, often comprising 10 items or less: for example, the Patient Health Questionnaire-9 (PHQ-9; Kroenke et al., 2001), the Generalized Anxiety Disorder-7 (GAD-7; Spitzer et al., 2006), the Clinical Outcomes in Routine Evaluation-10 (CORE-10; Barkham et al., 2013), Recovering Quality of Life-10 (ReQoL-10; Keetharuth et al., 2018), or the Short Warwick-Edinburgh Mental Wellbeing Scale (SWEMWBS; Stewart-Brown et al., 2009).

A separate line of activity within psychological therapy research has been the attempt to agree a core outcome battery in which researchers, in addition to selecting specific outcome measures appropriate for their particular study, adopt a single common outcome measure in order to make direct cross-study comparisons (e.g., Waskow & Parloff, 1975). Such an aspiration can then also be extended to cross-country and cross-cultural comparisons. But agreeing on a common measure carries many challenges and also places an additional burden on patients and on individual research studies, as well as requiring agreements between researchers from multiple countries and treatment concepts.

## Establishing the European Psychotherapy Consortium (EPoC)

Partly in response to these two lines of activity, the overarching framework underpinning the current project was establishing the European Psychotherapy Consortium (EPoC) with the initial aim of coordinating a level of standardized data collection across multiple European countries. In doing so, the aspiration was to generate data sets of considerable size, accessible to researchers that could add a new dimension to research findings and thereby extend our current knowledge base regarding the psychological therapies. In particular, such a development could provide researchers who have difficulty in securing funding, with access to a valuable data source to pursue their particular research projects.

The idea of creating EPoC was launched at the Rome meeting of the European Chapter of the Society for Psychotherapy Research (EU-SPR) in September 2022 with the aim of promoting the collection and sharing of data that is common in other sciences (e.g., physics, medicine, genetics) with the aspiration of improving research data in clinical psychology and psychological therapies in four specific ways. First, it would be relevant to psychological therapy research to have data from different countries, representing different cultural, political, and socio-economic realities. Second, it would facilitate cooperation between European countries, with enough data that could be used to better understand psychotherapy in naturalistic clinical settings. Third, in the long term, if the consortium were to be successful in collecting sufficient data, it may have some influence on decision-making processes at the individual patient level as well as on the implementation of psychological therapy at the mental health service level. Fourth, the consortium could facilitate the construction of large datasets, with considerable diversity (e.g., clients, diagnoses, cultural backgrounds, therapists), which would enable better possibilities for research on topics relevant to both practitioners and researchers as well as for cross-validation and replication of research findings.

## Aim

The immediate task within EPoC was to identify the measures used in different services within and across member countries. Our hypothesis was that there would be great heterogeneity in the measures used within and across countries, making it difficult to initiate international research collaborations. If this were indeed the case, our second task would be to find a pragmatic first-step solution to establish a common research ground for our international collaboration. As a consequence, it needed to be a measure that made the least demands on services, but which had sufficient face validity and psychometric credibility to be acceptable to members. In addition, services in multiple countries would have to be willing to voluntarily adopt it and administer it multiple times during treatment in order to build an international common research database.

A decision was taken to focus on the adoption of a single-item measure as the least burdensome method for clinics and services. To support this agenda would require a level of cross-country co-ordination and co-operation via the establishment of a virtual organization (i.e., EPoC). Accordingly, the current article sets out the organizational deliberations and actions to progress collaborations between psychological therapy researchers and practitioners across Europe with the initial aim of adopting a common single-item patient outcome measure.

## Method

The initial task at the beginning of the project was to establish the range of outcome measures used and to capture some of the key features of the way practice has been implemented in different contexts. A survey of EU-SPR members was the selected method of data gathering combined with an invitation to join EPoC.

### Part 1: Survey on Practice-Based Evidence

#### Survey Design

In addition to obtaining identification of a service, the survey was designed to capture basic information on four main areas relating to the functioning of a clinical service: (1) the setting and service provision, (2) the clinical populations served, (3) size/volume/throughput, and (4) the range of outcome/process measures used. Table 1 lists the 10 questions addressing these four areas.

**Table 1:** Question Topics in the EPoC Survey

Question topic	
1.	Identification data (e.g., country, type of service)
2.	Treatment options (e.g., outpatient, inpatient)
3.	Therapy models used (e.g., psychodynamic, cognitive-behavioural)
4.	Treatment modality (e.g., individual, couples)
5.	Patients under treatment (e.g., diagnoses)
6.	Estimates of number of patients per year and average number of sessions per patient
7.	Pre-post treatment measures used
8.	Outcome and process measures used at each session
9.	Other process and outcome measures that are used at regular intervals
10.	Whether there was routine outcome monitoring in the clinic

#### Procedure

The survey opened 1^st^ February 2023 and was advertised on the SPR mailing list, at scientific meetings, and on the EU-SPR website. The initial stock take of responses was carried out 31^st^ January 2024, thereby yielding data for a period of 12 months.

### Part 2: Selection of a Single-Item Outcome Measure

#### Status of Single Item Measures

As making significant changes to the instruments used in each clinic would be at best, challenging and at worst, impractical, a decision was made to propose introducing a single common item to be adopted by all participating clinics, translated in the language of each participating country. This was judged to be the minimal demand to achieve the maximum extent of possible participation by individual clinics. Historically, single-item measures have not been viewed in the most positive light. However, recent research has re-evaluated the evidence, which appears much more favorable (e.g., Ahmad et al., 2014). In addition, a recent editorial set out a ‘call to action’ regarding the adoption and testing of single item measures in psychological science (Allen et al., 2022). Hence, our strategy is consistent with such an agenda. Similarly, Vitry et al. (2024) have endorsed the rationale for single-item measures, and suggest they are particularly suited to psychotherapy patients and repeated measurements due to their low level of burden.

In terms of our method for the selection of a measure, we set two criteria. First, that the measure comprised a patient-completed item that captured the general psychological state or health of a patient. This criterion therefore excluded the Global Assessment of Functioning (Aas, 2010) and the Clinical Global Impression (CGI; Guy, 1976) scale. Second, that the selected measure utilized a Likert scale as we considered this to be easier to adopt, initially, in clinics as it did not require a subsequent stage of transferring the visual analogue scale into a numerical value. This criterion excluded consideration of the ORS, which actually comprises 4 items derived from the OQ-45 and, while there is considerable psychometric data reported on the ORS, a recent review offered some caution regarding its use (Harris et al., 2019).

## Results

The results are presented in two parts. First, we summarize the data relating to the clinical activity and use of outcome measures. Second, we present the selection of a single-item outcome measure for adoption across all participating clinics.

### Part 1: Survey of Clinical Activity and Outcome Measures

Responses were received from 31 clinics in 16 different countries, most of them European: Austria, Belgium, Finland, Germany, Hungary, Israel, Italy, Poland, Portugal, Slovenia, Spain, Switzerland, Turkey, United Kingdom, as well as Argentina and Chile. The estimated total number of clients per year was 25,000, with a median of 100 and a range from 12 in receipt of couple therapy to 15,000 per clinic. In terms of treatment settings, 31 were outpatient of which 10 were private, 3 were day clinical treatment, 3 were inpatient, and 1 was home-based treatment.

Of the 20 differing modalities of psychological therapy offered, the two most common were cognitive-behavioral therapy (*n* = 15) and psychodynamic (*n* = 14), followed by social-cognitive transactional analyses (*n* = 5), and then systemic, family therapy, and person-centered with each receiving 2 endorsement, and the remaining 11 endorsements captured single entries for the following therapeutic modalities: Mindfulness-Based Therapy, Solution / focused, Dynamic-Interpersonal Therapy, Brief relational, Eye Movement Desensitization and Reprocessing, Dialectical-Behavioral Therapy, Mentalizing model, Schema therapy, Schema group therapy, Cognitive Behavioral Analysis System of Psychotherapy, and Interpersonal Psychotherapy.

In terms of outcome data, 25 reported they were currently collecting data utilizing a total of 87 different pre-post measures, 22 measures used at each session, and 13 process measures. The most common pre-post outcome measure was the Clinical Outcomes in Routine Evaluation−Outcome Measure (CORE-OM; Evans et al., 2002), which was used in 13 clinics, the PHQ-9 (Kroenke et al., 2001) and GAD-7 (Spitzer et al., 2006) in 8 clinics, ANINT-A36 (Scilligo, 2000) and Espero (Scilligo et al., 1999) in 5 clinics, OQ-45 (Lambert et al., 1996) in 4 clinics, a further five measures were used in 3 clinics, 12 measures were used in 2 clinics, and the remaining 63 outcome measures used in only a single clinic.

In terms of outcome monitoring measures, the most common measure was the Outcome Rating Scale (ORS; Duncan & Reese, 2015), used in three clinics, with the OQ-10 (Lambert et al., 2005) used in 2 clinics. A further 19 measures were used in single clinics. When considering process measures, the most common was the Working Alliance Inventory–Short Revised (WAI-SR; Hatcher & Gillaspy, 2006), used in three clinics, with the Bern-Post Report (Flückiger et al., 2010) and the SRS (Duncan & Reese, 2015) used in 2 clinics. Five further measures were used in single clinics.

### Part 2: Selection of a Single-Item Outcome Measure: Emotional and Psychological Outcome (EPO-1)

In response to the survey, we focused solely on available single-item measures meeting our two election criteria. Our scoping of available single items identified one taken from the work of Ken Howard, for which a first version can be found in Orlinsky and Howard (1986), and has been adapted and employed successfully in several large-scale studies (e.g., Howard et al., 1996). The item was adapted and asks clients to evaluate their current emotional and psychological impairment using the question: “At this moment, how well do you feel you are getting along emotionally and psychologically?”. Based on the original item, the item is scored on a 5-point scale from 0 ("Very poorly; I can barely manage to deal with things") to 4 ("Very well; I have no important complaints"). The item can also be used dimensionally with a visual analog scale (0 to 100), which was introduced at the outpatient clinic of the University of Trier (Lutz et al., 2019). Hence, it provided the possibility of using a visual analog scale at a future date.

Robust correlations with various outcome measures have been demonstrated on a clinical sample (*N* = 521) with the correlations for the single item in both Likert and analog forms with the PHQ-9, the BSI depression and anxiety scales, OQ, and GAD-7 at baseline, for pre-post change, and overall effect size, exceeding those of the ORS 4 item total score (Supplemental Materials in Lutz et al., 2021). These data indicate that the single item has the potential to establish a common standard across diverse societies.

In order to make the single item identifiable in the literature, we took the focus on the *emotional* and *psychological* components (EP) together with *outcome* (O) and signified the single item by the digit (*1*); hence the name EPO-1.

#### Program of European Translations

EPoC members developed a narrative description of the content of the item (“lay description”) and a translation process for the item based on current best-practice recommendations (Table 2; e.g., Hernández et al., 2020) that required the following: active participation of members of the target population represented in the local setting (both clinicians and clients), and which offered good resource use for the purpose of translating one item. In addition to the English version, there are versions of the item translated into Finnish, French, German, Hungarian, Italian, Polish, Portuguese as well as Slovenian, and EPoC members are now involved in translating and adapting the item into Hebrew, and Spanish. This process results in a total of 11 language versions.

**Table 2:** Steps of the EPoC Translation Process for the Item

1.	Translation of the lay description of the item content provided by the consortium (to be used as supporting resource in the following process).
2.	Forward translation of the item and the response anchors by a team.
3.	Backward translation to English from the previous step, by a different team.
4.	Evaluation of the translation by practitioners (if the translation resulted in multiple possible versions, these would all be evaluated).
5.	Evaluation of the translation by clients (if the translation resulted in multiple possible versions, these would all be evaluated).
6.	Development of a final version based on results from Steps 3-5 by the local team (potentially including EPoC members in the discussions).
7.	Approval of final version by EPoC, licensing, and documentation on consortium’s web page.

#### Implementation and Dissemination

We have placed no restrictions on the use of the item, as some clinics may use it in every session, and others at regular intervals. Hence, the item will be adopted such that it is consistent with the current practice of each clinic. The item translations will be freely available and under a Creative Commons license after a free registration on the website of the European Chapter of the Society for Psychotherapy Research (item available at https://www.psychotherapyresearch.org/page/SPR-EU-Consortium).

## Discussion

We have set out the rationale and aims of a European-wide collaboration aimed at providing a common thread by which to yield a fuller understanding of the similarities and differences between the practices and outcomes of psychological therapies across multiple countries. Importantly, these are initial steps, achieved with no external grant funding by virtue of a shared vision to build a more robust and representative evidence-base for routine practice. In addition, this practical approach, focusing on a single item in a first step, allows routine clinics with diverse treatments and clinical populations to easily adopt ongoing monitoring in addition to established pre-post assessments.

Future collaboration will involve developing standardized reporting strategies (Snyder et al., 2019) and crosswalks between different measures (Schalet et al., 2021). The practical advantage of generating crosswalks is that it will enable a level of comparison between clinics and countries where different outcome measures are used. This could be at the level of individual scores or banding of scores signifying, for example, differing severity levels. From an organizational perspective, the availability of crosswalks means that clinics are able to select, within reason, their preferred outcome measure (i.e., protecting the principle of choice) but still be able to make direct comparisons (i.e., benchmark) with other clinics using different outcome measures, providing there is an existing crosswalk.

One way to do this is to focus on standardizing at least the underlying metrics of the instruments, to ease interpretation, for example by using *t* *scores* as a method of delivering uniformity to the reporting of outcomes from the diversity of measures (e.g., see de Beurs et al., 2022 for an illustration). Another solution is based on Item Response Theory: to develop an algorithm based on existing data with which values from different instruments that record similar constructs can be converted into each other. In this way, a common metric for existing data is generated a posteriori (e.g., Böhnke et al., 2014; Cardace et al., 2022; Schalet et al., 2021; Wahl et al., 2014). Other methods have also been used to deliver crosswalk tables (de Beurs et al., 2022), for example, between the BDI and CORE-OM (Leach et al., 2006).

So, the next step would be to create analytical routines among EPoC members that would allow comparison of similar constructs (e.g., depression, anxiety) despite using different instruments to measure them. A final future course of collaboration involves the creation of new datasets that could be articulated from the onset.

EPoC has colleagues with considerable experience of collecting data in routine care, and this offers the opportunity to create a task force that could be employed when new clinics want to start collecting data and have no external constraints on the instruments they need to use. This could be the starting point for the collection of more common measures. In fact, the survey also revealed that 23 additional clinics would like to start collecting new datasets, which would make it possible to introduce more common instruments that would allow a more direct comparison of measures.

Currently, the reality for the vision focuses on multiple clinics across differing countries harmonizing their data and is an initial step. However, it is likely that the greater challenge will arise with aspirations for data sharing. Hence, initial outputs from EPoC are likely to be locality specific with sharing occurring at the level of outputs or latent variables representing a common metric from crosswalk calculations and not primary data, thereby remaining within the existing agreements regarding patient consent and ethical approvals. It is now a priority to determine the scope, and likely hurdles, for data sharing in the future.

### Conclusion

Efforts to improve the effectiveness of psychotherapy require an understanding of the complex interplay between therapeutic interventions and the needs of individual clients in real-world settings. The launch of this project hopefully will mark a pivotal moment in collaborative psychotherapy research and practice in Europe. Bringing together committed researchers and practitioners from across Europe (and, in time, other countries), EPoC aims to harness the potential of assessments in routine care to provide a more nuanced understanding of psychotherapy in the setting in which it naturally occurs. With the potential to include a range of service settings, EPoC also offers an opportunity to improve our understanding of the implementation of systems for routine outcome monitoring, both on the organizational as well as on the concrete technical level (Böhnke & Rutherford, 2021).

Its ambition extends beyond the present to a future in which collaborative efforts produce large, accessible datasets that can inform service delivery both at a local, national, and international level. The participation of colleagues from Latin America in EPoC will enhance collaboration with the Latin American Chapter and is a first step toward data collection collaboration between different continents. The aim is to address specific issues of practice-based evidence in different regions, for example, regarding implementation but focusing on the same long-term goals. This scientific endeavor invites therapists to join forces in a collective quest to advance research on psychological therapies, contribute to a growing body of knowledge, and thrive in a community that shares insights and is committed to unraveling the intricacies of effective clinical practice.

## Supplementary Materials

EPoC – European Consortium of Psychotherapy – is a group closely associated with the Society for Psychotherapy Research (SPR). The list of members that make up this group is available in a supplementary file (see Gonçalves et al., 2024S).



GonçalvesM. M.
LutzW.
SchwartzB.
OliveiraJ. T.
SaarniS. E.
TishbyO.
RubelJ. A.
BoehnkeJ. R.
MontesanoA.
PaivaD.
CeridonoD.
ZechE.
WillemsenJ.
SaarniS. I.
Kompan ErzarK.
JaneiroL.
GeloO. C. G.
ErrázurizP.
HolasP.
StyłaR.
RožičT.
RosenströmT.
BékésV.
UnokaZ.
BarkhamM.
 (2024S). Supplementary materials to "Developing a European Psychotherapy Consortium (EPoC): Towards adopting a single-item self-report outcome measure across European countries"
[Additional information]. PsychOpen. 10.23668/psycharchives.15263
PMC1163674439678318

## Data Availability

The data that support the findings of this study are available on request from the corresponding author, João Tiago Oliveira. The data are not publicly available due to containing information that could compromise the privacy of research participants.

## Social Media Accounts

@jtcruzoliveira

## Reporting Guidelines

We followed principles of good and open reporting in the absence of any specific guideline.
